# Part orientation optimization for Wire and Arc Additive Manufacturing process for convex and non-convex shapes

**DOI:** 10.1038/s41598-023-29272-x

**Published:** 2023-02-07

**Authors:** Yazan Alomari, Márton Tamás Birosz, Mátyás Andó

**Affiliations:** Faculty of Informatics, Eötvös Loránd University, Savaria Institute of Technology, Szombathely, Hungary

**Keywords:** Engineering, Mechanical engineering

## Abstract

Building orientation optimization for Additive Manufacturing (AM) process is a crucial step because it has a vital effect on the accuracy and performance of the created part. Wire and Arc Additive Manufacturing’s (WAAM) working space is less limited, and the production time is significantly shorter than the other metal 3D printers. However, one of the adverse effects of WAAM is the defect at the start and endpoints of the welding beads. In this paper, an algorithm has been invented to define the optimal printing position, reducing the number of these defects by rotating the 3D object in a loop around the X and Y axes by a small constant degree and then selecting the degree of rotation that has the fewest uninterrupted surfaces and the largest area of the first layer. The welding process will be interrupted as little as possible by the torch if there are the fewest possible uninterrupted surfaces. As a result, there will be fewer defects in the production and finishing of the welding beads. In order to have a sufficient connection surface with the build tray, which will aid in holding the workpiece in place, the largest first layer should also be sought. Therefore, it has been found that a properly defined orientation relative to the build tray can reduce the number of uninterrupted surfaces within the layers, which will improve the expected dimensional accuracy of the parts. The efficiency of the process is highly affected by the shape of the part, but in most cases, the print errors can be drastically minimized.

## Introduction

In recent years, as Additive Manufacturing (AM) became a popular topic among industry and academic researchers, many development directions have been launched from different disciplines. Manufacturing engineers and machine designers develop newer solutions for additive layer-by layer production. According to the specific need, such as the minimum production time, volume, and accuracy^1–4^ they create subtypes processes such as Selective Laser Sintering (SLS) for creating high precision metallic parts, Fused Deposition Modeling (FDM) for creating cheap plastic products and wire arc additive manufacturing (WAAM) which has a great advantage in producing large-size structures. Simultaneously, material scientists created a diverse range of raw materials that can be used for manufacturing^3,5^. As a result, robust concrete printers, machines capable of working with conductive raw materials, or even biological fabrics can be found on the market. Designers develop their shape optimization methods utilizing the freedom of 3D printed geometries, respecting the inhomogeneous anisotropic mechanical behavior and other aspects arising from the layered structures^6,7^. Furthermore, since AM perfectly meets the requirements of Industry 4.0, several researches focus on creating intelligent manufacturing systems, incorporating IoT devices, and enhancing the usage of CAD-CAM systems^8–14^.

The most important factor for each above-mentioned discipline is understanding the boundary conditions and limits of the technologies. In contrast to traditional subtractive methods, additive methods have different technological characteristics. One of the main tasks for producing an AM part is to find the perfect printing orientation. With this one setting, many manufacturing technology problems can be eliminated, and the end-product properties can be greatly determined with it. Shim et al.^15^ They investigated the printing accuracy, mechanical properties, and surface characteristics of the parts printed in different orientations and found the optimal settings as follows: with a layer thickness of 100 m, they printed the parts in 3 different printing orientations (0, 45, and 90 degrees). According to their analysis of the results of the final printed parts, the specimens printed at 0 degrees had the highest flexural strength, followed by specimens printed at 45 and 90 degrees. The specimens printed at 45 and 90 degrees showed the lowest error rates for length, and the specimens printed at 0 degrees had the highest error rates for thickness. Alharabi et al.^16^ examined the effect of the printing orientation and consequently the direction of the layers under compression test. They found that if the layers are perpendicular to the load direction it has the higher compressive strength, than parallel. The surface roughness as a function of build direction was investigated by Li et al.^17^. They concluded that this property is mainly affected by the build angle rather than by the AM method, and the best surface roughness can be achieved on the faces that are printed parallel or perpendicular relatively to the build platform. Pandey et al.^18,19^ worked on minimalizing these effects by creating a system that predicts the surface roughness mathematically utilizing the multicriteria genetic algorithm and offers the best print orientation for Fused Deposition Modeling (FDM), the benefit of this solution is that they obtained the optimal surface roughness orientation; however, they did not consider all the limitations factors of the 3D printing and manufacturing process. Additionally, machine learning (ML) models are a new modeling trend in AM. Fundamentally, ML models work on the tenet of iteratively reducing expected error using data. They have shown to be reliable predictive tools. Xia et al.^19^ modeled and forecasted the surface roughness of metal produced by wire arc additive manufacturing using machine learning methods. Phatak and Pande^20^ also created an optimization solution using a genetic algorithm to minimalize the machining time and surface errors. The generic algorithm was used in the study made by Masood et al.^21^, to find the best orientation for complex shaped parts. With their developed system, they were able to determine the best orientation where the overall volumetric error is the minimum. Padhye et al.^22^ used Multi-objective Optimization and Multi-criteria Decision Making to determine the optimum considering two factors, print time and surface roughness. Their work points out that decision-making becomes more complicated when the orientation has to be satisfied by several aspects at the same time. Furthermore, Morgan et al.^23^ developed software specifically to minimize the support requirements for metal additive manufacturing. Therefore, based on the research in these articles, it can be said that a seemingly insignificant setting, such as orientation, can have a significant impact on the quality of the production in several ways, such as accelerating the printing process, lowering surface roughness, or improving mechanical properties.

**Figure 1:**
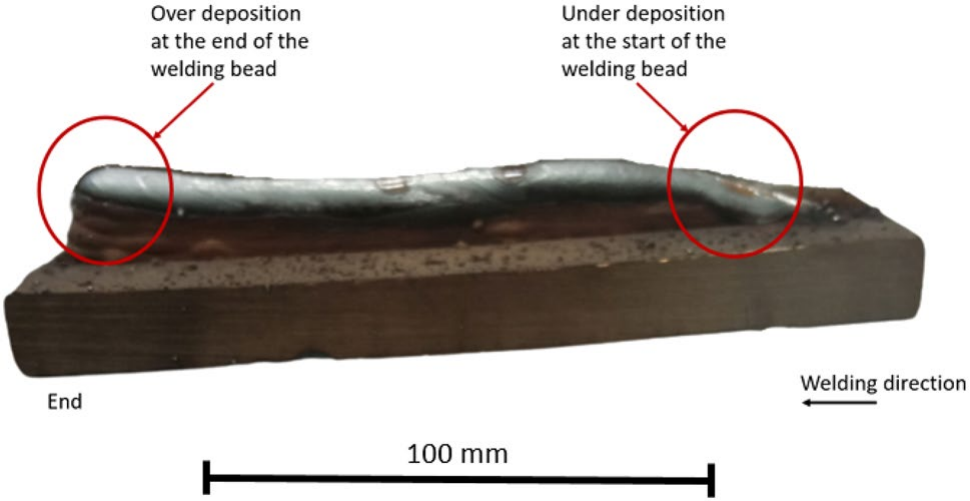
Shape Forming Problem of the Welding Process.

One of the novel AM techniques is Wire and Arc Additive Manufacturing (WAAM), which answers the problem of most AM solutions by providing a rather fast manufacturing time and allowing the creation of large volume metal parts^24^. However, Fig. 1 illustrates a shape forming problem that happens at the start and end of the welding process. This article demonstrated how by choosing the appropriate print parameters, this issue could be solved.

Locket et al.^25^ concluded the criteria that should be followed. According to them, the guidelines are: central web on a part plane of symmetry, planar outer and inner walls, and the plane of symmetry or partial symmetry. These are the fundamental aspects, but some imperfections resulting from the welding process remain even by designing the part according to them. In most related research works^26,27^ an outstanding difference can be observed in the bead geometry. Due to the arc forming, the start and endpoints of the beads have a slightly bigger or smaller local volume than the areas in-between the ends. Another remarkable peculiarity is the anisotropy inside the printed part^28^. As the additive process creates the layered part, hard and soft regions alternately can be observed in the number of layers respectively. Both adverse effects can be reduced by an appropriate print orientation selection. Thus, in this paper, a part optimization method is presented, which aims to minimize the printing imperfections of parts created by Wire and arc Additive Manufacturing (WAAM). Moreover, the presented algorithm can be used to any other deposition-based methods, but the presented problem and its solution are most pronounced with WAAM technologies. A MATLAB code has been created, and a parameter sensitivity check has been carried out to investigate the performance of the presented solution.

## Theory

### WAAM starting problem

The WAAM is a 3D printing solution that suffers from weakness to initialize and finish a single welding bead. In the beginning, the welding torch approaches the build plate (or the previous) layer until the feedstock material reaches the surface when the arc is created, and the welding operation begins. The process after this is similar to the other extrusion-based AM technologies. The layers are created by “drawing” the layers by the welding “line”. Due to the delayed responses of the welding instrument, which creates the necessary electric conditions for the welding, and the robot which is responsible for moving the torch, the very first and the end segment of the beads always show some imperfections. Thus, at these locations, geometrical inaccuracies and surface errors can be observed. This inaccuracy can be observed on the parts created by other deposition based 3D printing technology, where a layer has to be “drawn”. Furthermore, if this geometrical defect is located at the same location in multiple players, it may result in a packed error and can ruin the whole print. Also, the layered structure shows anisotropic behavior, which is most considerable along the build direction, i.e., between two layers. Both problems can be solved with special welding solutions, such as crater filling or offsetting the start and endpoints. However, some dimensional accuracy and microstructural imperfections will always be present. As it cannot be fully eliminated, the secondary guideline would be to minimize the number of local errors. Therefore, the continuity of welding must be ensured as much as possible by creating one long weld in the printed layer instead of many smaller sections. For simple geometries, it can be done by following a spiral trajectory, Hilbert curve, or filling out the layer with a zig-zag path. Where for more complex components, due to the interrupted surfaces, it is not always possible. In general, increasing the surface area of each layer as well as reducing the number of layers, the need for initializing a new bead can be significantly reduced.

### STL file

**Figure 2:**
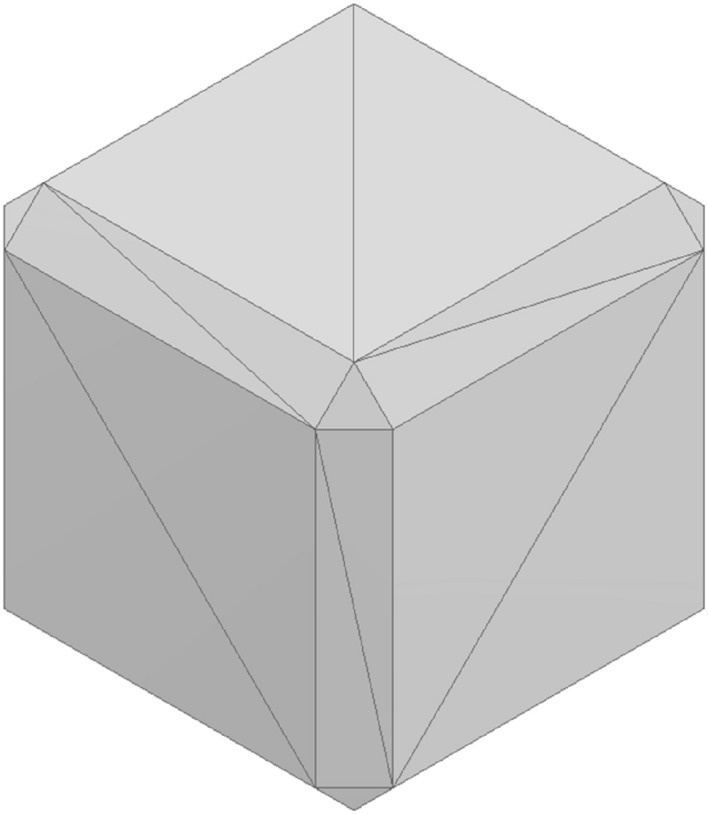
STL representation of cube with fillets on the edges.

AM systems are based on a toolpath generated via slicing software, where the model geometry has to be imported, and the printing parameters have to be set. The model geometry is usually created in Computer Aided Design (CAD) software or is mapped from a 3D scan as reverse engineering. In both cases, the file format should be a Standard Triangle Language (STL) file, which is used for most 3D printing solutions^29^. This format gives the surface model of the virtual geometry by dividing the surface of a body into many interconnected triangles. The generated file includes each triangle's vertex, as seen in Fig. 2, and may subsequently be readily edited.

### Rotation

**Figure 3:**
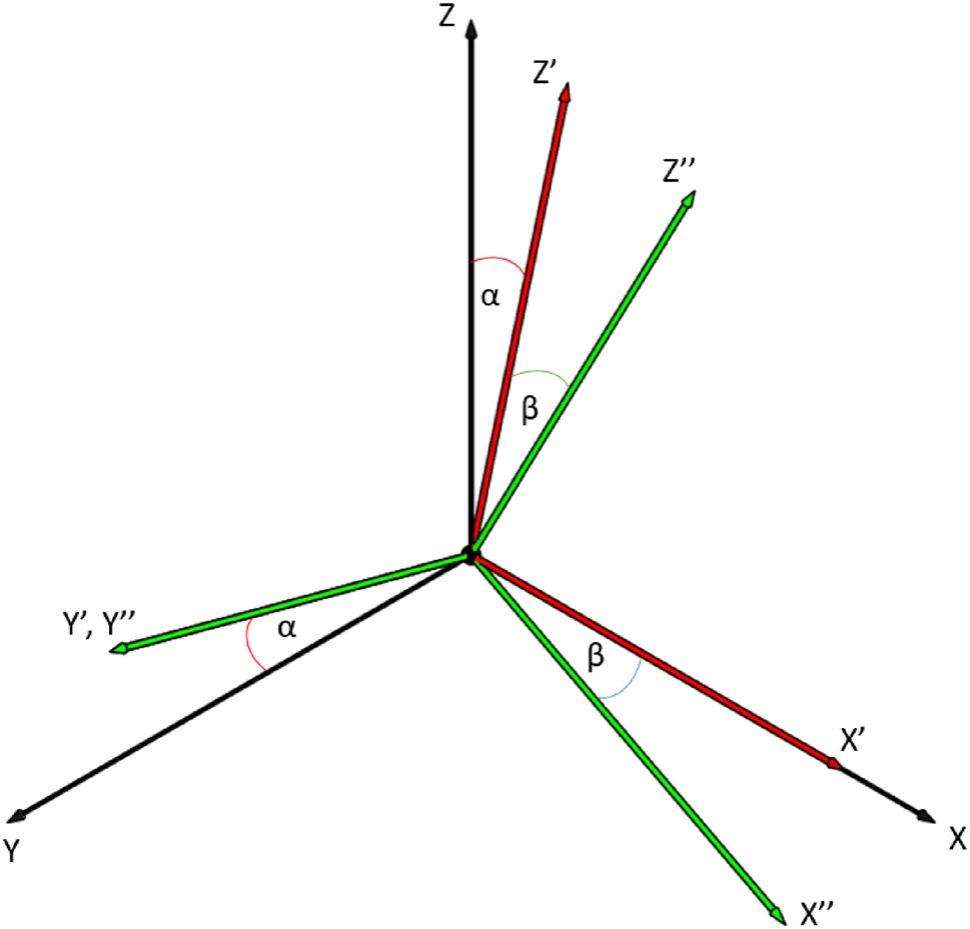
Euler transformation.

To find the best orientation for a part, which fulfils the above-detailed criteria, the model of the part has to be rotated in space. Here, the well-known Euler transformation was used^30^. With this, the orientation of any rigid body can be described to the initial fixed Descartes coordinate system. The process consists of three consecutive elementary rotations according to the Proper Euler angles (z–x–z, x–y–x, y–z–y, z–y–z, x–z–x, y–x–y) or the Tait-Bryan angles (x–y–z, y–z–x, z–x–y, x–z–y, z–y–x, y–x–z). Since the angular position normal to the build plate does not affect in this case, the rotation along this axis, namely the Z-axis in this study, is irrelevant. Thus only two rotations are required (x–y or y–x). The representation of the Euler transformation in a positive direction, respectively to x–y order can be seen in Fig. 3. It can be seen that after the first rotation around the X axis the direction of the new Y’ and Z’ directions have been changed by α angle compared to the originals (Y and Z). Meanwhile the X’ direction remained the same (X). After the second rotation, this time around the Y’ axis of the previously created coordinate system, the direction of X’’ and Z’’ have been changed by β angle, while Y’’ remained the same (Y’). It can be concluded that in this way every printing orientation can be checked with a given angle resolution.

The resulting rotation matrix is the following (1,2):

1 $$ \vec{R} = \left[ {\begin{array}{*{20}c} 1 & 0 & 0 \\ 0 & {{\text{cos}}\left( \alpha \right)} & { - {\text{sin}}\left( \alpha \right)} \\ 0 & {{\text{sin}}\left( \alpha \right)} & {{\text{cos}}\left( \alpha \right)} \\ \end{array} } \right]\left[ {\begin{array}{*{20}c} {{\text{cos}}\left( \beta \right)} & 0 & {{\text{sin}}\left( \beta \right)} \\ 0 & 1 & 0 \\ { - {\text{sin}}\left( \beta \right)} & 0 & {{\text{cos}}\left( \beta \right)} \\ \end{array} } \right] $$

2 $$ \vec{R} = \left[ {\begin{array}{*{20}l} {{\text{cos}}\left( \beta \right)} \hfill & 0 \hfill & {{\text{sin}}\left( \beta \right)} \hfill \\ {\left( { - \sin \left( \alpha \right)} \right) \cdot \left( { - \sin \left( \beta \right)} \right)} \hfill & {{\text{cos}}\left( \alpha \right)} \hfill & {\left( { - \sin \left( {\upalpha } \right)} \right) \cdot {\text{cos}}\left( {\upbeta } \right)} \hfill \\ {{\text{cos}}\left( \alpha \right) \cdot \left( { - \sin \left( \beta \right)} \right)} \hfill & {{\text{sin}}\left( \alpha \right)} \hfill & {{\text{cos}}\left( \alpha \right) \cdot {\text{cos}}\left( \beta \right)} \hfill \\ \end{array} } \right] $$

The position of each investigated instance can be expressed with a single vector using this transformation, and the vertices of the STL file can be altered accordingly. The next step is to determine the best rotation vector that fits all the mentioned conditions in “WAAM starting problem” section. As a result, the part geometry must be sliced at each rotated position in order to obtain the layer boundaries. However, to determine the distance between the layers, the layer thickness should be set based on what the manufacturing technology can produce. The slices can be interpreted from the intersections of the object surface and the plane that can be formed from the offset of the X–Y plane at the investigated layer height.

**Figure 4:**
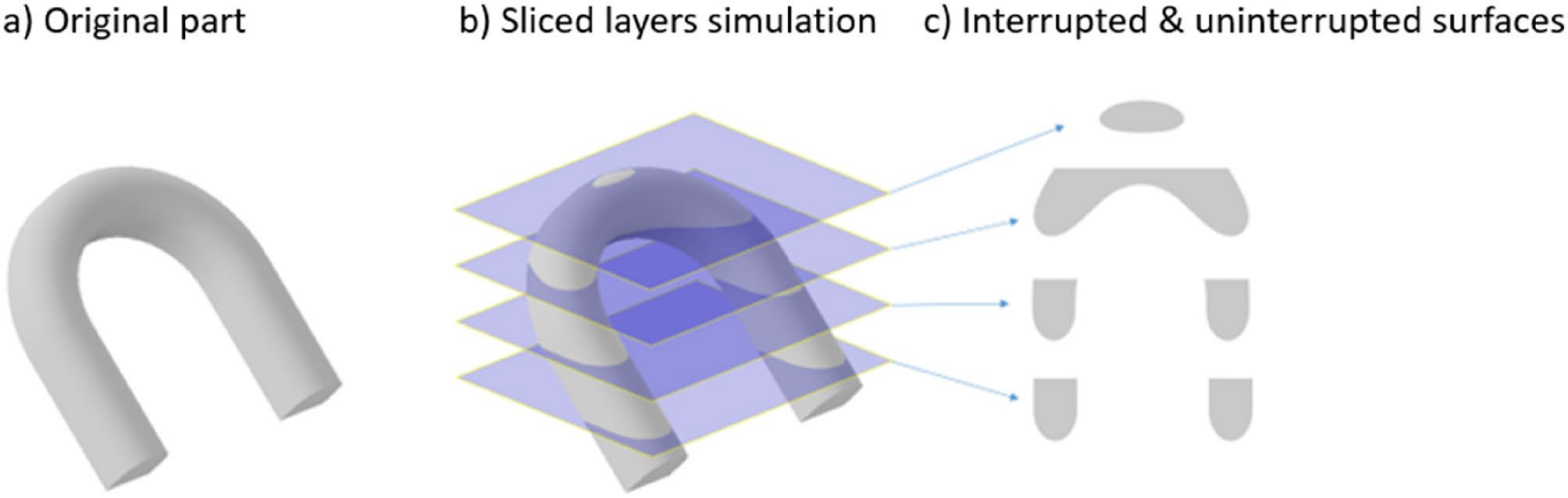
Representation of the meaning “uninterrupted surfaces” on the layers.

The difference between the concept of individual uninterrupted surfaces and the layers number can be seen on Fig. 4. As it is highlighted, depending on the complexity of the geometry, there can be several separate surface elements withing one layer. However, this articulation can be affected by print orientation, that is, how each layer slices the part.

### The proposed algorithm

In the proposed method, the 3D object is rotated in a loop around the X and Y axes by a small constant degree until it reaches 180 degrees on both axes. In each rotation, the number of uninterrupted surfaces and the area of the first layer is calculated and stored along with the regarded X and Y axes degrees.

**Figure 5:**
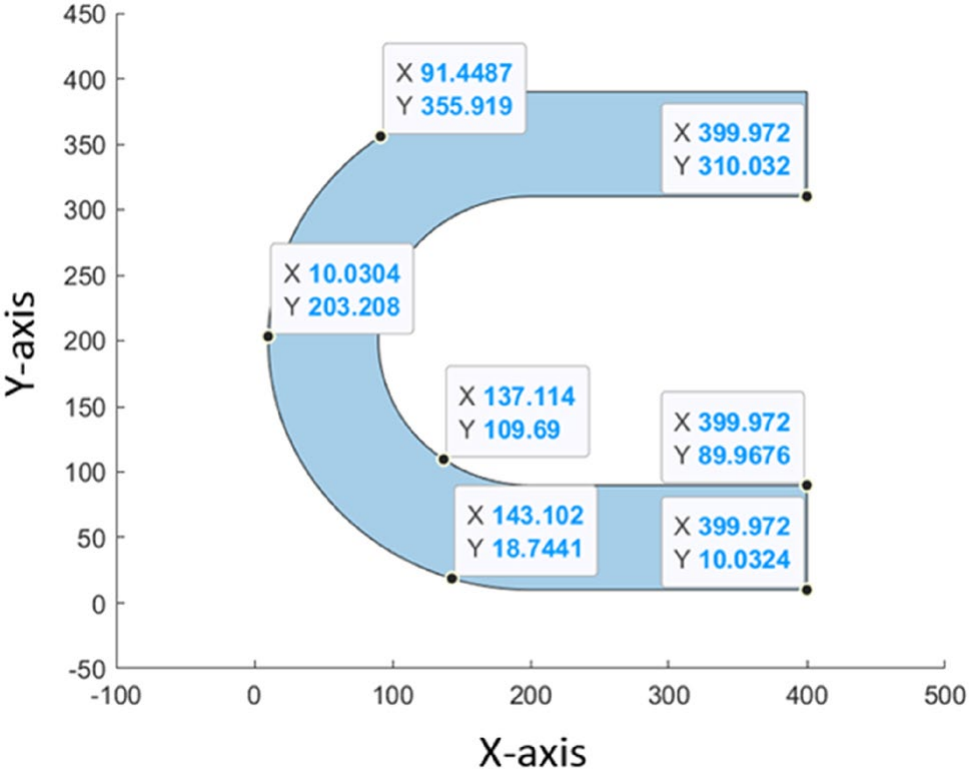
Sample of layer with some of it’s 2-D verticies.

Calculating the area of the layers is highly challenging, especially in the case of non-convex shapes. To address this issue, we proposed the following accurate technique: It starts by reading the binary stl file and then creating triangles, which are then sliced into layers with pre-defined heights to provide a list of coordinates. To begin, we must first locate the triangles that intersect the slicing plane. Then, using a graph depth-first search (DFS) algorithm, it computes the lines generated on the triangle face by the intersection and creates a continuous path connecting the lines. Figure 5 depicts a slice and some of its coordinates. As a matter of fact, each slice is a polygon defined by 2-D vertices. The surface area of a 2-D polygon is defined by the vertices in the vectors x and y.

**Figure 6:**
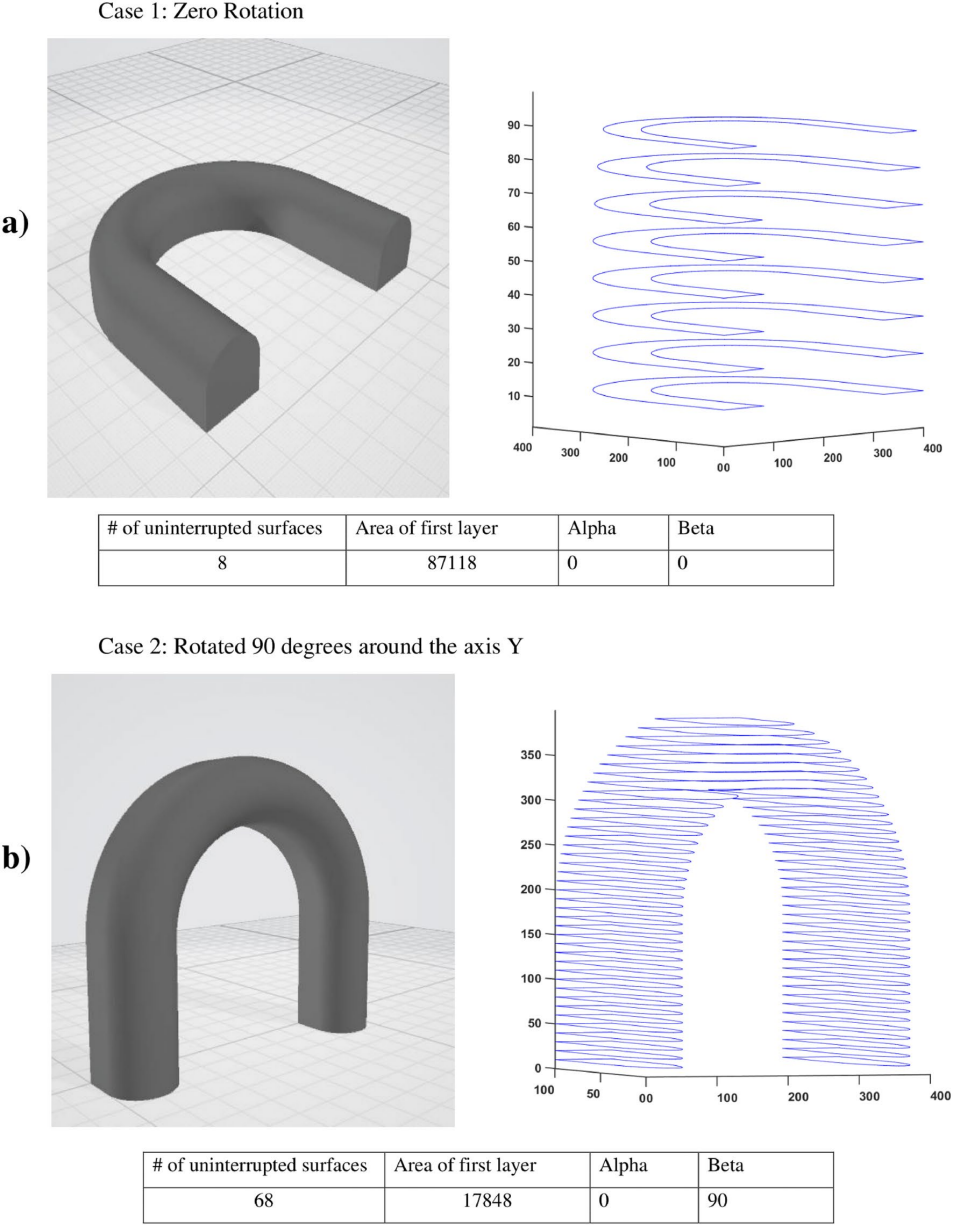
Representation of slices of a 3D object (**a**) laid, and (**b**) upright.

The difference between the two placements of the same 3D object is shown in Fig. 6a,b; the object is lying horizontally on the bed in the first scenario. The object was sliced with a slice height of 10 at that point, resulting in 8 layers with no interrupted surfaces. The object has been rotated 90 degrees around the axis Y in the other scenario, and sliced using the same settings, resulting in 39 layers with 68 uninterrupted surfaces.

Finally, the rotation degree that has the least number of uninterrupted surfaces and the largest area of the first layer is selected. The lowest number of uninterrupted surfaces means that the torch must interrupt the welding process the least possible, so the number of defects in the formation and completion of the welding beads can be minimized. In addition, the proper adhesion of the first layer is necessary to prevent the deformation of the part during the printing process or the actual breaking of the part. Therefore, the largest first layer should be pursued to have a large enough connection surface with the build tray, which will help to hold the workpiece in place. The proposed algorithm is according to the Table 1.

**Table 1:**
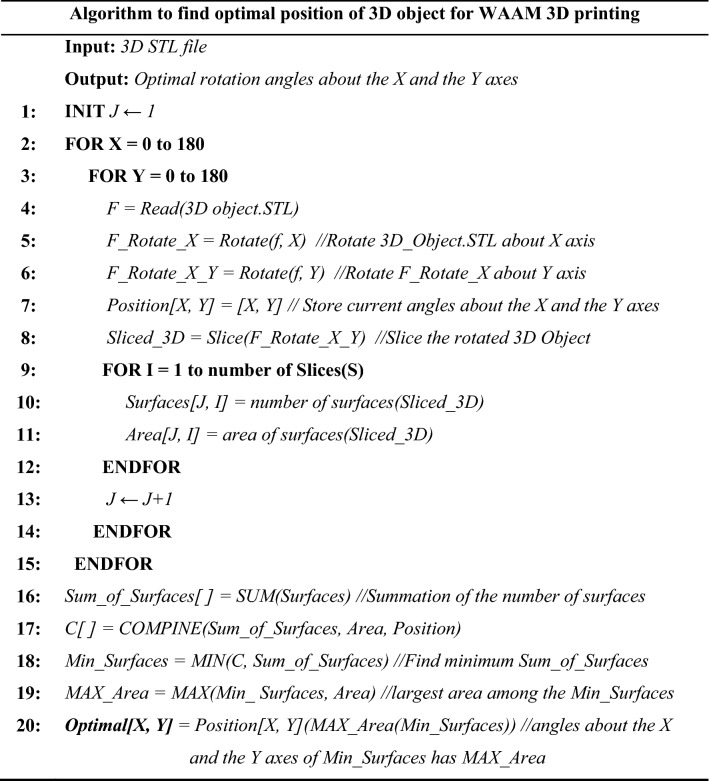
Algorithm.

### Tested geometries

**Figure 7:**
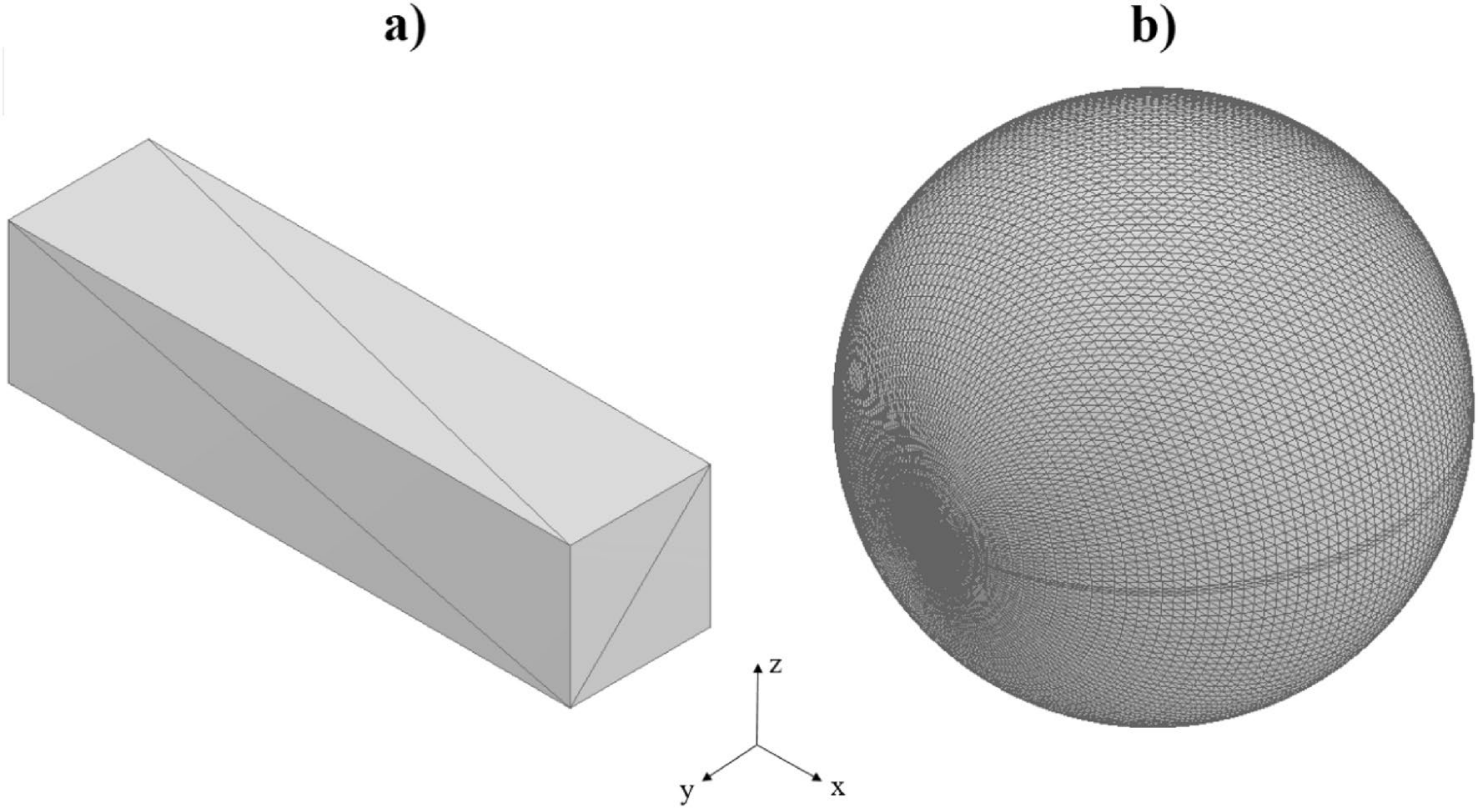
Convex shapes (**a**) square-based prism and (**b**) sphere.

Common engineering practice is to build up a part as a combination of representative shape primitives, such as spheres, blocks, cubes, cylinders, toruses, etc. Therefore, as an initial investigation of the software function, the shape primitives, shown in Fig. 7, were tested. To create these shapes Autodesk Inventor Professional 2018 has been used, and the files have been saved in .stl format, which was read by the Matlab algorithm.

**Figure 8:**
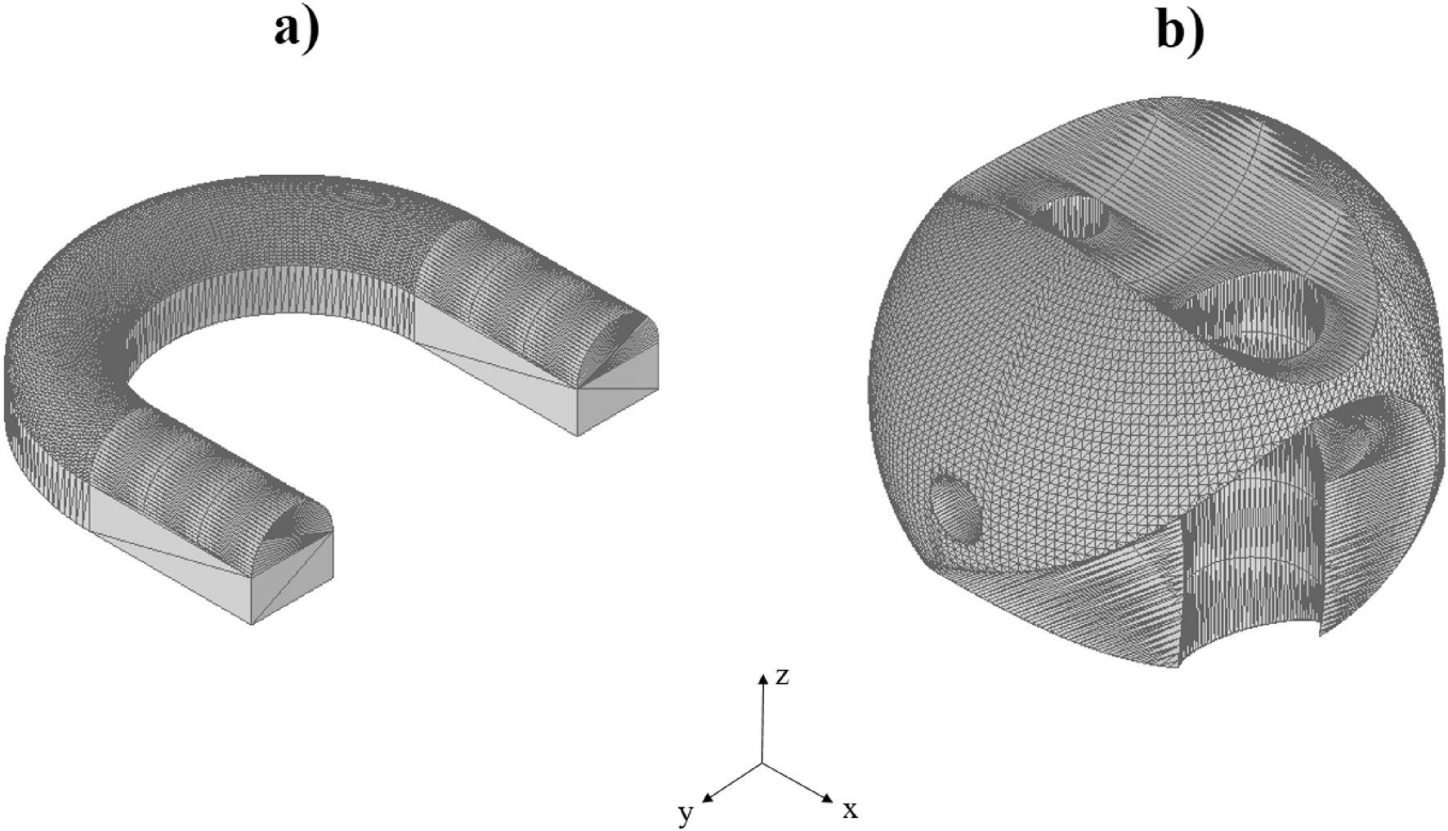
Non-convex shapes (**a**) U-shape and (**b**) special sphere.

These shapes (Fig. 7) are all convex, which means that every line inside the shape can be connected, while this line will remain inside the shape. However, in some cases, in more complex parts some sliced layer will contain multiple unconnected surfaces. Therefore, the algorithm is be able to handle the non-convex shapes as well (Fig. 8).

## Results and discussion

The developed algorithm was applied to the four shapes presented in the previous section. The initial orientation is presented in Figs. 7 and 8, and the corresponding angles are with respect to these coordinate systems. The convex shapes (cubic prism and sphere) can be considered as a test operation since the outcome can be predicted without using the created Matlab code. Multiple positions were found to be best build orientations for the Prism, with the least number of uninterrupted surfaces, as indicated in Table 2, where α and β are angles of rotation around the X and Y axes. Only the lowest number of uninterrupted surfaces is displayed in the table; the other rotations resulted in a larger number of uninterrupted surfaces. The geometry, in this case has 3 symmetry planes. Therefore, each rotated position has 6 equivalents.

**Table 2:** Number of surfaces in the prism.

α / β	0	90	180
0	99	99	99
180	99	99	99

The sphere has an infinite symmetry plane. Table 3 shows the achieved result, which was the same in all conceivable orientations, indicating that the number of total surfaces is the same regardless of orientation, implying that no optimal orientation can be discovered. These two simple forms (prism and sphere) can be considered as test elements of the algorithm, as the number of layers and surfaces associated with each orientation in each layer can be easily estimated by the user as well.

**Table 3:** Number of surfaces in sphere.

α / β	0 …180
0 …180	399

For the non-convex shapes, first, the U-shape was investigated. From the results provided in Table 4, it can be seen, that rotating around the X and Y-axis increases the number of surfaces which must be created during the printing process. Four optimal orientations can be found for this geometry, that are similar to each other, just like in the case of the prism. However, among these only two meets the secondary optimization condition, explained later in this section.

**Table 4:** Number of slices in U-shape.

α / β	0	180
0	98	98
180	98	98

Lastly, the special truncated sphere has been checked. This is a representative part, which would be significantly expensive to manufacture with traditional subtractive machining, thus a sufficient representative of the capabilities of WAAM process. Due to the more complex geometric features that make up the body, it would be quite a challenge to state the correct printing position based on purely the user’s decision. The results gave only one optimal orientation as presented in Table 5, which fulfils the primary condition.

**Table 5:** Number of slices in the special sphere.

α / β	30
40	406

**Figure 9:**
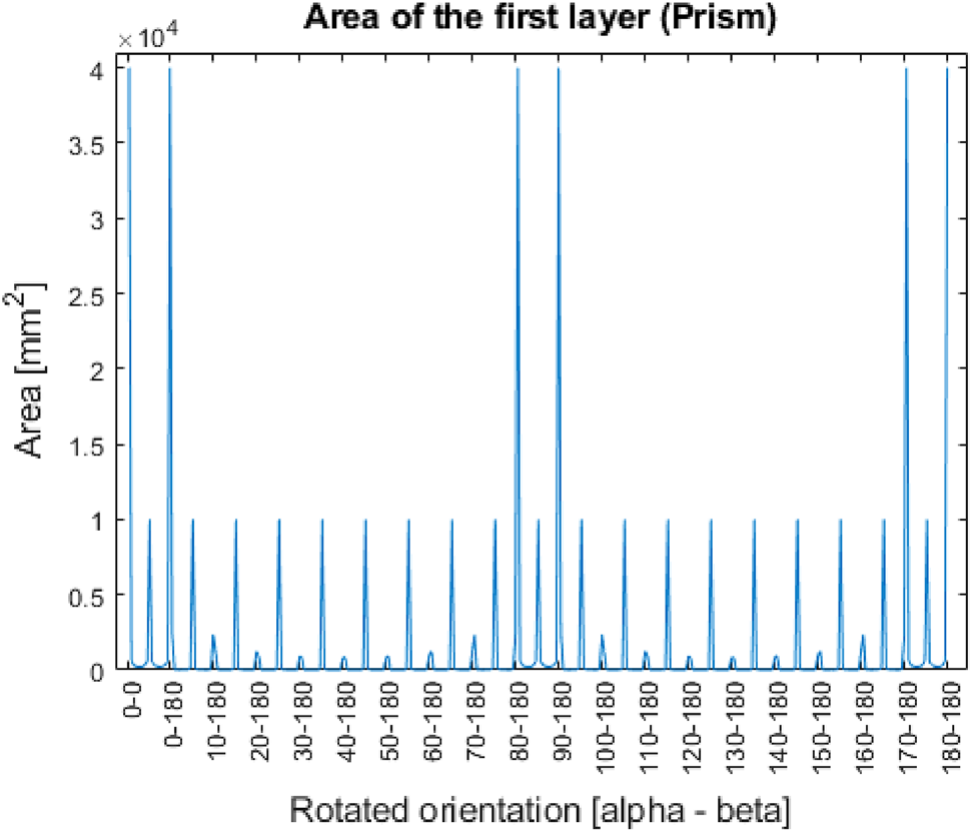
Area of the first layer (Prism).

**Figure 10:**
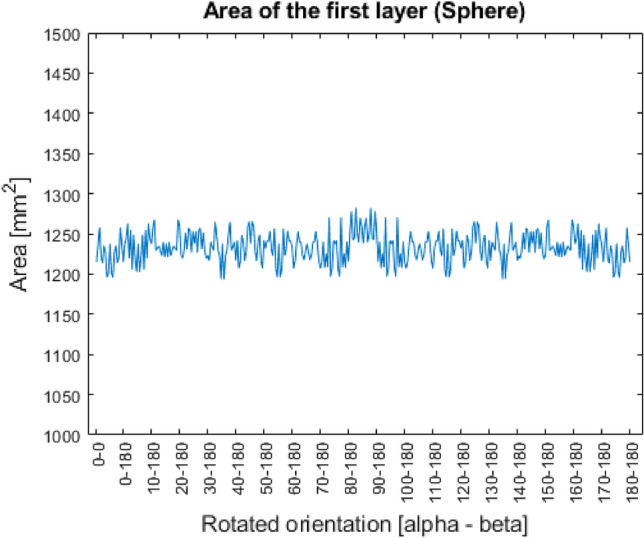
Area of the first layer (Sphere).

**Figure 11:**
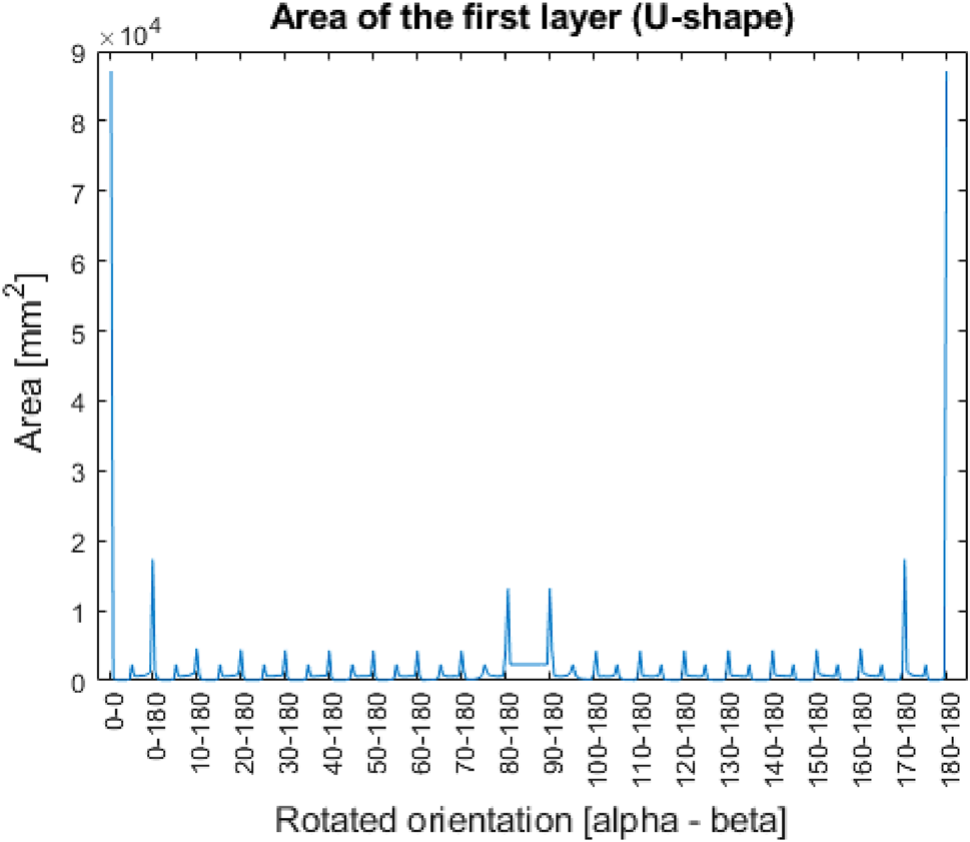
Area of the first layer (U-shape).

**Figure 12:**
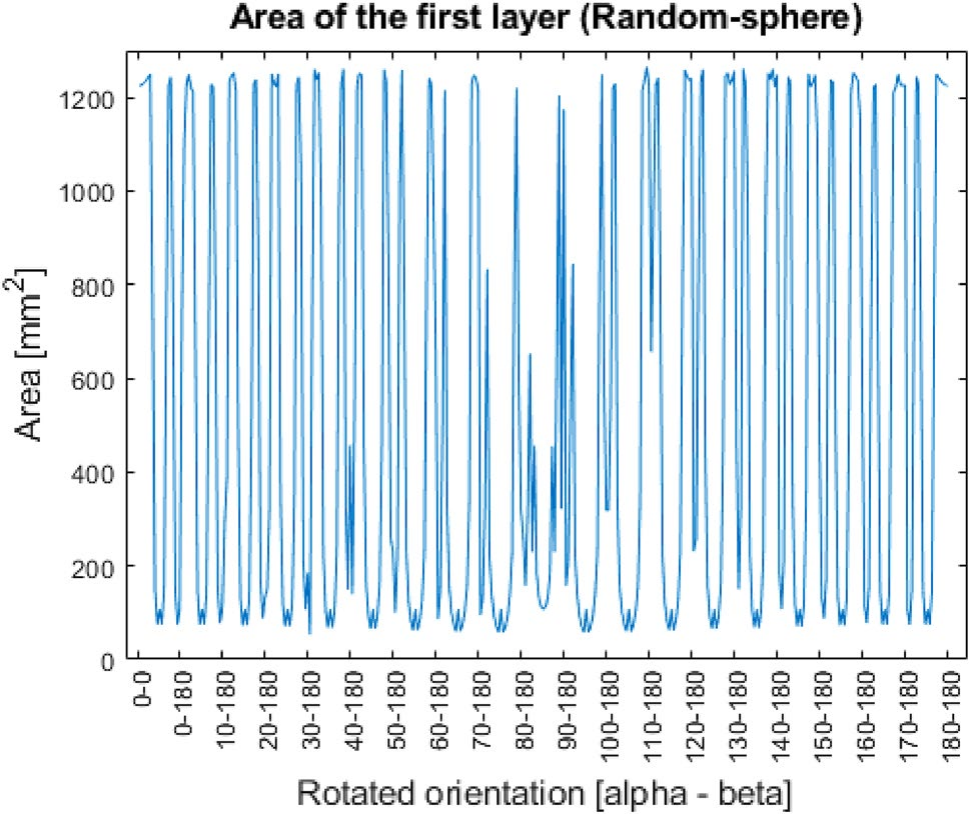
Area of the first layer (special sphere).

As for the secondary optimization condition the area of first layers has been investigated. The results for the four shapes are presented in the Figs. 9, 10, 11, 12.

In the Figs. 9, 10, 11, 12, it can be seen that in case of three out of four shapes some patterns were created. Since every geometry except the special sphere has one or more symmetry planes, the same area can be measured in different rotated orientations, since they mean the identical orientation from the point of view of the investigation.

As it was expected, for the prism the 6 best orientations according to the primary optimization condition meets with the 6 biggest first layer area. Therefore, it can be stated that these positions are equally the best possible, as they are identical for the study.

In case of the sphere shape, in theory, there shouldn’t be any difference regarding the first layer. However, because of the resolution of the STL file, in some positions the enveloping tringle may be located normal to the print direction, which result as a slightly bigger layer area. The magnitude of the deviation is not significant, and since in reality a perfect sphere would connect to the build tray only at one point, the value of the first layer’s area can be neglected. Here, it’s worth to mention, that after the welding process the part must be machined off from the build tray via some chipping technology, so to ensure the safety of the manufactured product, some support-like structure must be placed between the part and the tray. The concept of the maximal first layer area for optimization only aims to provide to add an additional guide for the designer. The importance of this condition can be considered by the user.

As it can be seen in Table 4, the U-shape gave 4 identical optimal position, and by assessing with the areas, it can be limited into two. The 0–0 and the 180–180 orientations both lie on the bottom flat surface as can be seen on the Fig. 6.

The results of the Special-sphere shape offer another approach to evaluate and determine, what we call optimal orientation. Here, the minimum number of surfaces is not associated with the maximum first layer area. In terms of proportions, the difference is not significant, but it can be assumed that for some highly complex shapes the establishment can be problematic. The following Eq. (3) can help the user to determine, which is the more need condition for optimization.

3 $$ {\text{max}}\left( {O_{{\left( {alpha, beta} \right)}} } \right) = \left( {w \cdot A_{{first \left( {alpha, beta} \right)}} } \right) - \left( {\left( {1 - w} \right) \cdot N_{{surf\left( {alpha, beta} \right)}} } \right) $$

where *O*_*(alpha, beta)*_ is the rotated orientation, *A*_*first*_ is the area of the first layer, *N*_*surf*_ is the number of the uninterrupted surfaces, and *w* = (0,…, 1) is a constant value, that can be set by the user based on their preference. For instance, if the user assumes that the area of the first layer has not a great importance, the value of w can be chosen as a relatively bigger number and the algorithm will mainly focus on to find the orientation where the number of surfaces is the minimal.

The acquired optimal orientation, as demonstrated in the results and explanation, would produce the best minimal value of the number of individual surfaces, resulting in less welding deficits, as shown in Fig. 1. The gap between the achievable minimum and the worst-case situation, which is the maximum, is, nevertheless, greatly dependent on the geometry's complexity. In the case of a sphere, for example, each printed orientation will produce the same outcome because the object has an endless number of symmetric projections, but in the case of the special sphere, even a slight difference in angle might significantly affect the produced number of individual surfaces.

## Conclusion

An algorithm has been developed to determine the most optimal print orientation to minimize WAAM-specific manufacturing defects. The investigation was performed on the STL file of some representative part geometries, and for creating the algorithm MATLAB software has been used. At the start and endpoints of the welding beads, a significant geometrical inaccuracy, and mechanical property defect can be observed such as inhomogeneous hardness and tensile properties within the part. As it is inherent in the technology, this effect cannot be fully eliminated but can be minimized. This can be achieved by appropriately selecting the build orientation of the geometry regarding the printing process. In this article, the proposed algorithm was able to find the optimal build orientation with very low complexity as well the following two parameters were looked at in priority order:

1. Using the proposed approach, the printer is about to manufacture fewer uninterrupted surfaces, so the chance of such a geometrical deficiency occurring is decreased.
2. In the case of multiple optimum findings, the one which has the maximal initial layer should be selected, thus a sufficient build plate connection can be achieved.

## Data Availability

The datasets used and/or analyzed during the current study available from the corresponding author on reasonable request.
